# Mediators of physical activity change in a behavioral modification program for type 2 diabetes patients

**DOI:** 10.1186/1479-5868-8-105

**Published:** 2011-09-29

**Authors:** Delfien Van Dyck, Karlijn De Greef, Benedicte Deforche, Johannes Ruige, Catrine E Tudor-Locke, Jean-Marc Kaufman, Neville Owen, Ilse De Bourdeaudhuij

**Affiliations:** 1 Department of Movement and Sport Sciences, Ghent University, Watersportlaan 2, 9000 Ghent, Belgium; 2 Research Foundation Flanders (FWO), Belgium; 3 Department of Human Biometry and Biomechanics, Vrije Universiteit Brussel, Pleinlaan 2, 1050 Brussels, Belgium; 4 Department of Endocrinology, Ghent University Hospital, De Pintelaan 185, 9000 Ghent, Belgium; 5 Walking Behavior Laboratory, Pennington Biomedical Research Center, 6400 Perkins Road, Baton Rouge, LA 70808, USA; 6 Baker IDI Heart and Diabetes Institute, Melbourne; The University of Queensland, Brisbane, Australia

## Abstract

**Background:**

Many studies have reported significant behavioral impact of physical activity interventions. However, few have examined changes in potential mediators of change preceding behavioral changes, resulting in a lack of information concerning how the intervention worked. Our purpose was to examine mediation effects of changes in psychosocial variables on changes in physical activity in type 2 diabetes patients.

**Methods:**

Ninety-two patients (62 ± 9 years, 30, 0 ± 2.5 kg/m^2^, 69% males) participated in a randomized controlled trial. The 24-week intervention was based on social-cognitive constructs and consisted of a face-to-face session, telephone follow-ups, and the use of a pedometer. Social-cognitive variables and physical activity (device-based and self-reported) were collected at baseline, after the 24-week intervention and at one year post-baseline. PA was measured by pedometer, accelerometer and questionnaire.

**Results:**

Post-intervention physical activity changes were mediated by coping with relapse, changes in social norm, and social modeling from family members (p ≤ 0.05). One-year physical activity changes were mediated by coping with relapse, changes in social support from family and self-efficacy towards physical activity barriers (p ≤ 0.05)

**Conclusions:**

For patients with type 2 diabetes, initiatives to increase their physical activity could usefully focus on strategies for resuming regular patterns of activity, on engaging family social support and on building confidence about dealing with actual and perceived barriers to activity.

**Trial Registration:**

NCT00903500, ClinicalTrials.gov.

## Background

Epidemiological data consistently link increased physical activity to reduced mortality risk in type 2 diabetes patients [1]. Despite the established benefits [2], many type 2 diabetes patients do not participate in regular physical activity [3]. This highlights the need to develop efficacious physical activity interventions for this particular patient group [4].

We developed a behavioral modification program to increase physical activity in type 2 diabetes patients [5]. Since effective behavioral modification programs are necessarily based on established correlates, it is needed to take theoretical models into account when developing an intervention. This intervention was based on constructs from the social cognitive theory [6], the transtheoretical model [7] and the self-determination theory [8]. Constructs derived from these theories have been widely accepted to understand and promote physical activity [9-13], both in general populations and type 2 diabetes patients. Based on the consistent associations with physical activity, the following theory-based constructs were targeted in the intervention: modeling, social norm, social support, self-efficacy, benefits, barriers, coping with relapse, processes of change and motivation. The intervention itself consisted of an individual face-to-face session by a psychologist, the use of a pedometer and a 24-week schedule of follow-up telephone support (by the psychologist), including topics on social support, self-efficacy, benefits, barriers, decisional balance, goal-setting, problem-solving strategies, time management, coping with relapse and motivation. The intervention aimed at gradual increases in physical activity, starting from the participants' baseline levels. The protocol and content of the intervention have been described in detail elsewhere [5]. This behavioral modification program showed positive effects on steps/day, accelerometer-based and self-reported physical activity over the short-term and intermediate term [5].

Most studies, including our own, reported the behavioral impact of physical activity interventions, but few studies have reported changes in theoretical constructs preceding behavioral changes or examined possible mediators, resulting in a lack of information concerning how the intervention worked [14-17]. Results from earlier studies in a general population are mixed but the most common mediators of intervention effects on physical activity seem to be behavioral processes (substituting alternatives, enlisting social support, rewarding yourself, committing yourself and reminding yourself) and self-efficacy [16,18,19]. For decisional balance and social support, mixed results were found [19-21]. Mediators of intervention effects in type 2 diabetes patients have been rarely studied. Barrera and colleagues [22,23] investigated social support (from family, friends and neighborhood) as a short- and long-term mediator and only found a short-term effect. Dutton and colleagues [24] found that self-efficacy completely mediated physical activity among type 2 diabetes patients after a brief one-month intervention period.

To better understand which variables mediate physical activity improvements in a type 2 diabetes population, additional research utilizing prospective and controlled trials is needed [24-26]. Mediators should be examined at multiple time points, including both short-term and long-term time points [19,24] and objective measures of physical activity should be used [24].

We examined whether the effects of a physical activity program were mediated by the theoretical constructs targeted by the intervention, both post-intervention (after 24 weeks) and at one year. It was hypothesized that self-efficacy and social support (derived from social cognitive theory) would be changed by the intervention and that these changes would mediate the changes in self-reported and objective physical activity, as has been previously demonstrated [24]. As the intervention was also based on the self-determination theory and the transtheoretical model, the other theoretical constructs targeted (e.g. motivation, coping with relapse, modeling) were also examined as potential mediators of change.

## Methods

### Participants and procedure

The study protocol is described in detail elsewhere [5]. A sampling pool of potential participants was generated from the Endocrinology Department of the Ghent University Hospital in Belgium. The inclusion criteria were: 1) ≥ six months post-diagnosis of type 2 diabetes; 2) age: 35-75 years; 3) body mass index (BMI): 25-35 kg/m^2^; 4) treated for type 2 diabetes; 5) no documented physical or medical physical activity limitations 6) Dutch speaking; 7) having a telephone number, and 8) having a follow-up appointment with their endocrinologist during the recruiting period from July till December 2007. Based on these criteria, a total population of 143 individuals were identified as eligible to participate and invited by mail to participate in the study. Thirty-two showed no interest, two s passed away prior to the study and 17 could not participate because of medical reasons. The remaining 92 agreed to participate in the study and were called to be enrolled. They were subsequently randomly assigned to an intervention (n = 60) or a control group (n = 32) using an imbalanced randomization 2:1. Every participant signed an informed consent form. The non-stratified randomisation was performed using sealed envelopes so the group allocation was concealed until the point of allocation. Blinding to group allocation could not be maintained post-recruitment, as with most behavioral interventions. The psychologist did the blinded group allocation, as well as the measurements, the intervention and the statistical analyses.

Three one-week assessments were spread over one year: at baseline, immediately after the 24-weeks intervention (post-intervention) and one year after baseline. The measurement one year after baseline was called 'intermediate-term' as it was not considered sustainable enough to speak about long-term changes. For the assessments, all participants were visited at home. During this visit, the International Physical Activity Questionnaire (IPAQ) was completed by interview. During the week following the home visit, participants were asked to complete a questionnaire on psychosocial correlates of physical activity, to wear an accelerometer and a pedometer, and to record their pedometer steps/day in a logbook. The Ethical Committee of the Ghent University Hospital approved the study.

### Measures

#### Sociodemographics

The basic information on age, weight, height, diabetes duration of the sample was retrieved from the patient files, and from a sociodemographic questionnaire that was filled out by the participants.

#### Objective and self-reported physical activity measurements

Physical activity was measured using a pedometer (steps/day), an accelerometer (min/day) and the IPAQ (min/day). The pedometer (Yamax DigiWalker SW200, Tokyo, Japan) and the accelerometer (Actigraph, model 7164) were worn at the waist during waking hours for seven consecutive days. Both the pedometer and accelerometer are valid and reliable tools used to objectively measure physical activity [27,28]. An activity log was used to record the steps taken, and the type and duration of non-walking activities [29]. For every minute of non-walking activities (cycling or swimming) reported, 150 steps were imputed at the end-day number of steps [29,30]. The outcome variables of the accelerometer were time spent at activities of different intensity [31]. For the present analyses, accelerometer-based total physical activity (= light-intensity physical activity + moderate-intensity physical activity + vigorous-intensity physical activity) was used as outcome variable. The long IPAQ Dutch interview version was used to assess self-reported physical activity. The interview version was chosen as our previous experiences showed that the self-report version lead to many unanswered items in the questionnaires and massive over-reporting [32]. The interview version was administrated 3 times in every participant in the same standardized way, by the same researcher, but with special attention to specific explanations for seniors and with special attention to decrease overreporting following a standardized protocol. Validity and reliability of the interview version of the long IPAQ have been shown to be acceptable in a 12-country study [33]. In the questionnaire, frequency (number of days) and duration (hours and min/day) of physical activity in different domains (work, transportation, leisure time, housekeeping and gardening) were queried. Minutes/week of physical activity in the different domains was calculated by multiplying frequency by duration.

#### Psychosocial correlates

As the intervention was based on theoretical constructs from the social cognitive theory [6], transtheoretical model [7] and self-determination theory [8], all the different constructs were queried in the psychosocial questionnaire. More detailed information on the construction and content of the psychosocial questionnaire is given in Table 1.

**Table 1:** Structure and content of the psychosocial correlates included in the psychosocial questionnaire

Theory or model	Psychosocial construct/scale	Number of items	Chronbach's alpha	Example of item
Self-determination theory [8]	Amotivation	4	.83	I do not understand why I should do any PA
	External regulation	4	.79	I do PA because other people tell me that I have to
	Introjected regulation	3	.80	I feel guilty when I do not do PA
	Identified regulation	4	.73	I do PA because it is good for my overall health
	Intrinsic regulation	4	.86	I do PA because it is fun

Social cognitive theory [6]	Modeling from family	2	.78	How frequently do family members participate in PA?
	Modeling from friends	2	.71	How frequently do friends participate in PA?
	Modeling from general practitioner	1		How frequently does you general practitioner participate in PA?
	Social norm from family	2	.77	Do your family members think you should participate in PA?
	Social norm from friends	2	.75	Do your friends think you should participate in PA?
	Social norm from general practitioner	1		Does you general practitioner think you should participate in PA?
	Social support from family	2	.73	How often does your family invite you to do PA together with them?
	Social support from friends	2	.75	How often do your friends encourage you to be physically active?
	Social support from partner	2	.79	How often does your partner encourage you to be physically active?
	General self-efficacy	1		I think I can be regularly active
	Self-efficacy towards barriers of PA	16	.92	I think I can be physically active, even if I am not feeling well
	Perceived benefits: appearance	3	.65	Feeling more attractive
	Perceived benefits: psychological	5	.87	Feeling less tense and stressed
	Perceived benefits: health	7	.85	Improving my longs and the condition of my heart
	Perceived benefits: pleasure	3	.57	Having fun
	Perceived benefits: social	2	.67	Having the chance to meet new people
	Perceived benefits: diabetes-related	3	.81	Better monitoring of my diabetes

Transtheoretical model [7]	Perceived barriers: age-related	3	.82	I feel too old to do PA
	Perceived barriers: health	7	.90	Lack of good health (injury, sickness,...)
	Perceived barriers: psychological	6	.76	Having personal problems
	Perceived barriers: diabetes-related	5	.84	Fear of going into hypoglycemia when doing PA
	Perceived barriers: lack of interest	8	.80	Lack of interest in PA
	Perceived barriers: external	6	.82	Lack of PA facilities
	Coping with relapse	3	.80	Do you think you are able to make an inventory of high-risk situations that can contribute to relapse episodes?

*Note*: all items were rated on a five-point Likert scale except for self-efficacy towards barriers of physical activity (three-point scale) PA = physical activity

Motivation for physical activity (derived from the self-determination theory) was assessed using the Behavioral Regulation for Exercise Questionnaire (BREQ-2) [34]. This questionnaire was chosen because it specifically assesses motivation towards participation in physical activities. This is a validated questionnaire consisting of five scales: amotivation, external regulation, introjected regulation, identified regulation and intrinsic regulation.

Modeling, social norm, social support, general self-efficacy, self-efficacy towards barriers of physical activity, perceived benefits (outcome expectations) (all derived from the social cognitive theory), and perceived barriers towards physical activity and coping with relapse (derived from the transtheoretical model) were also assessed. Questions were selected and adopted from a previous study in adults [35].

Modeling was measured by asking participants how frequently their family, friends and general practitioner were physically active. Social norm was assessed by asking if their family, friends and general practitioner thought that they should be physical active. To investigate social support, participants were asked if they had a regular sport partner, how often their family, friends and partner invited them to exercise with them and how frequently they encouraged them to participate in physical activity.

The level of self-efficacy towards specific barriers was obtained by asking participants how confident they were that they can be physically active under 16 potentially difficult situations (early in the morning, depressive mood, family expectations, lots of work to do, not feeling well, end of a long tiring day, major life events, social obligations, etc.). General self-efficacy towards physical activity was also inquired.

Perceived benefits and barriers with regard to physical activity were investigated by asking respondents to rate their agreement with possible positive effects of physical activity (23 items) and the frequency with which barriers prevented them from exercising (35 items). Benefits and barriers were each divided in six subscales with good internal consistency, based on previous studies [35]. Coping with relapse, was assessed by asking participants if they thought they were able to make an inventory of future high-risk situations that can contribute to relapse episodes and cope with these situations.

### Statistical analysis

Data were analyzed using SPSS 15 with baseline carried forward intention-to-treat principles. Descriptive statistics of the study sample were analyzed and differences in baseline characteristics between the intervention group and the control group were examined using independent sample t-tests. In case of significant differences in baseline characteristics, these factors were included in the mediating analyses as confounding factors. Coping with relapse and changes in modeling, social norm, social support, general and specific self-efficacy, perceived benefits, perceived barriers, decisional balance, and motivation were examined as potential mediators of the intervention effects on changes in physical activity behavior (pedometer steps/day, accelerometer-based total physical activity, and self-reported active transportation, physical activity for housekeeping and gardening, leisure-time physical activity, and total physical activity).

Measures of change in physical activity behaviors between pre- and post-intervention test and between pre- and one-year follow-up test were created by regressing the physical activity measures at post-intervention test and at the one-year follow-up test onto their baseline values. Based on these regression outcomes, residualized physical activity change indices were computed. These scores can be interpreted as the amount of increase or decrease in physical activity behaviors between baseline and either subsequent time point, independent of baseline activity [36]. A similar measure of residualized change in psychosocial correlates (except for coping with relapse, for which only the post-intervention values and the one-year follow-up values were used) was created by regressing each psychosocial factor score at post-intervention test and at the one-year follow-up test into the baseline scores. These measures of change in psychosocial factors are also independent of baseline scores [36].

As suggested by Cerin and colleagues [37], the Freedman-Schatzkin difference-in-coefficients test was used to assess the mediating effects of the changes in psychosocial factors on the change in physical activity behaviors. This method was used instead of the traditional Baron-Kenny causal step approach, because the Baron-Kenny method has low statistical power in studies with a small sample size, even when strong mediating effects are present [37]. The Freedman-Schatzkin test measures a mediating effect by comparing the relationship between the independent (the intervention) and the dependent variable (change in physical activity behaviors) before and after adjustment for the mediator (change in psychosocial variables). For each potential mediator, this test was repeated (single mediation analysis). Using the Freedman-Schatzkin method, the null hypothesis that the difference between the unadjusted (without mediator: τ) and adjusted (with mediator: τ') regression coefficients of the independent variable is zero, was tested. The test consists of three regression analyses. The first analysis examines the impact of the intervention condition (dummy variable: 0 = control group, 1 = intervention group) on the outcome measure, providing an estimate for τ (relationship between intervention condition and physical activity behavior change before adjusting for the mediator). The second regression looks at the associations between the intervention condition (independent variable) and the potential mediators (dependent variables). This step is necessary because a significant intervention effect on the potential mediators is required to do mediating analyses [37]. The third regression analysis looks at the effect of the intervention condition on the outcome measure, after controlling for the mediator (residualized change in psychosocial factors), giving an estimate for τ' which represents the independent effect of the intervention condition on physical activity change after adjusting for the mediator. The significance test for the mediating effect is computed by dividing (τ - τ') by its standard error and comparing the obtained value to a t-distribution with N-2 degrees of freedom. If the t-value is > 1.984, there is a significant mediation effect at the 5% level [37]. The proportion of the intervention effect mediated by each psychosocial factor was calculated by subtracting the adjusted relationship between the intervention exposure and physical activity change (τ') from the unadjusted relationship (τ), and dividing the sum by the unadjusted value ((τ-τ')/τ) [38].

In all analyses, the total sample (both intervention group and control group; n = 92) was included. Statistical significance was set at p < .05. p-values between .10 and .05 were described as being marginally significant.

Based on intervention effects on number of steps/day in previous research [39], an a priori power analysis was conducted. Based on 0.80 power to detect a significant difference (p = 0.05, two-sided), 25 patients were required for each study group.

## Results

### Sample characteristics

Baseline sample characteristics of the demographic and psychosocial variables are presented in Tables 2 and 3. At baseline, the mean age of the participants was 62 ± 9 years and 69% were males. Mean BMI was 30.0 ± 2.5 kg/m^2^. The majority of the participants (82%) were diagnosed with type 2 diabetes more than five years previously and 44% received a combination of oral medication and insulin for their condition. There were no differences in descriptive, demographic and psychosocial characteristics at baseline between the control and intervention group, except for diabetes duration, introjected regulation, identified regulation, intrinsic regulation, social norm from general practitioner and general self-efficacy (all higher for intervention group). Since these differences might confound the results, the significant variables were included as confounding factors in all analyses.

**Table 2:** Sample characteristics of descriptive and demographic variables at baseline

Characteristic		Baseline characteristics	T-value
Age (years)	Intervention group	62.37 ± 9.25	.88
	Control group	60.59 ± 9.05	
Weight (kg)	Intervention group	89.22 ± 12.63	1.72
	Control group	84.50 ± 12.38	
BMI (kg/m^2^)	Intervention group	30.24 ± 2.62	1.07
	Control group	29.60 ± 3.02	
Diabetes duration (years)	Intervention group	11.87 ± 9.66	1.99*
	Control group	8.72 ± 5.50	
Steps/day	Intervention group	4959 ± 2414	-.32
	Control group	5139 ± 2933	
Total physical activity (min/day) (activity monitor)	Intervention group	300 ± 90	-1.03
	Control group	322 ± 109	
Active transportation (min/day) (self-report)	Intervention group	12 ± 19	.33
	Control group	11 ± 16	
Leisure time physical activity (min/day) (self-report)	Intervention group	17 ± 23	-.25
	Control group	19 ± 25	
Total physical activity (min/day) (self-report)	Intervention group	59 ± 60	-.02
	Control group	60 ± 59	

*p < .05

**Table 3:** Sample characteristics of psychosocial variables at baseline (n = 92) (mean (± SD))

		Baseline measurements	T-value
Amotivation	Intervention group	1.56 (0.83)	0.60
	Control group	1.66 (0.74)	
External regulation	Intervention group	2.23 (0.98)	0.62
	Control group	2.08 (1.20)	
Introjected regulation	Intervention group	2.67 (1.07)	2.26*
	Control group	2.11 (1.16)	
Identified regulation	Intervention group	3.45 (1.03)	3.28**
	Control group	2.67 (1.20)	
Intrinsic regulation	Intervention group	3.10 (1.15)	2.91**
	Control group	2.33 (1.28)	
Modeling family	Intervention group	2.08 (1.07)	0.97
	Control group	2.36 (1.32)	
Modeling friends	Intervention group	1.93 (1.09)	0.14
	Control group	1.97 (0.96)	
Modeling general practitioner	Intervention group	3.20 (1.27)	0.51
	Control group	2.90 (1.66)	
Social norm family	Intervention group	3.52 (1.21)	0.13
	Control group	3.48 (1.23)	
Social norm friends	Intervention group	2.57 (1.45)	0.07
	Control group	2.55 (1.17)	
Social norm general practitioner	Intervention group	4.52 (0.74)	2.54*
	Control group	4.00 (1.14)	
Social support family	Intervention group	2.00 (1.21)	0.44
	Control group	1.92 (0.90)	
Social support friends	Intervention group	2.05 (1.03)	0.45
	Control group	2.17 (0.93)	
Social support partner	Intervention group	2.43 (1.03)	0.07
	Control group	2.45 (1.21)	
Self-efficacy towards barriers	Intervention group	1.90 (0.44)	0.87
	Control group	1.81 (0.47)	
General self-efficacy	Intervention group	3.77 (0.87)	2.57*
	Control group	3.19 (1.18)	
Perceived benefits	Intervention group	3.62 (0.74)	0.67
	Control group	3.51 (0.85)	
Perceived barriers	Intervention group	2.52 (0.86)	0.19
	Control group	2.55 (0.75)	

*Note*: All items except for level of self-efficacy towards specific barriers of physical activity (1-3) had a five-point Likert scale (1-5).

*p < .05, **p < .01

Dropout during the 24-week intervention was 3.3% (two individuals in the intervention group lost interest and one individual in the control group was hospitalized). One year after baseline, dropout was 4.3% (one more individual from the control group became immobile).

### Changes in psychosocial factors as mediators of short-term (pre-post) intervention effects on physical activity outcomes (Table 4)

**Table 4:** Mediating effects on the short-term (pre-post) intervention effects on change in physical activity (PA) behaviors

	Steps/day	Self-reported active transport	Self-reported PA house + garden	Self-reported leisure-time PA	Self-reported total PA
τ (SE)	3642.70 (524.40)	98.46 (27.29)	163.97 (76.44)	119.80 (43.47)	336.11 (91.42)
p	<.001	.001	.035	.007	<.001

*Mediator: change in social norm from family*					
τ' (SE)	3466.71 (499.45)	79.92 (24.84)	147.59 (71.31)	118.77 (41.17)	311.31 (87.12)
p	<.001	.002	.042	.007	.001
τ - τ'	175.99	18.54	16.38	1.03	24.80
t	1.99*	3.35*	1.09	.12	1.41
Proportion mediated	4.8%	18.8%			

*Mediator: change in modeling from family*					
τ' (SE)	3612.85 (516.90)	90.51 (24.55)	146.19 (72.18)	105.11 (41.55)	311.39 (88.35)
p	<.001	.001	.046	.014	.001
τ - τ'	29.85	7.95	17.78	14.69	24.72
t	.30	1.41	1.20	2.07*	1.42
Proportion mediated				12.2%	

*Mediator: coping with relapse*					
τ' (SE)	3419.58 (516.77)	70.45 (25.11)	146.05 (73.57)	105.50 (42.41)	298.14 (89.00)
p	<.001	.006	.051	.015	.001
τ - τ'	223.10	28.01	17.92	14.30	37.97
t	2.36*	4.52*	1.06	2.05*	2.18*
Proportion mediated	6.1%	28.4%		11.9%	11.3%

*p < .05

τ = relationship between intervention condition and outcome measure before adjusting for mediator

τ' = relationship between intervention condition and outcome measure after adjusting for mediator

SE = standard error

*Note*: in all analyses, the total sample (n = 92, both control group and intervention group) was included

#### Step 1

After controlling for the confounding variables, the intervention was a significant positive predictor of short-term change in steps/day (p < .001), and the following self-reported physical activity variables: active transportation (p = .001), physical activity for housekeeping and gardening (p = .035), leisure-time physical activity (p = .007) and total physical activity (p = .044). The mediator-unadjusted τ-coefficients of the significant regression analyses are shown in Table 4.

#### Step 2

The intervention was a significant positive predictor of coping with relapse (β = .414; SE = .204; p = .046) and a marginally significant positive predictor of short-term change in modeling from family (β = .471; SE = .274; p = .086) and change in social norm from family (β = .528; SE = .305; p = .087). For the different types of motivation, modeling from friends and general practitioner, social norm from friends, social support, self-efficacy, benefits and barriers, no significant results were found (all p > .10). Therefore, only changes in social norm from family, modeling from family and coping with relapse were analyzed as potential mediators of the short-term (pre-post) intervention effects on changes in physical activity behaviors.

#### Step 3a - Mediating effects of change in social norm from family

After adjusting for change in social norm from family (Table 4), the intervention condition remained a significant positive predictor of change in steps/day (p < .001) and change in self-reported active transportation (p = .002), but the adjusted regression coefficients (τ') were significantly lower than the unadjusted τ-coefficients (t = 1.99 and t = 3.35). Thus, the short-term increase in social norm from family mediated the intervention effect on steps/day (4.8%) and the intervention effect on self-reported active transportation (18.8%).

Change in social norm from family was not a significant mediator of the short-term intervention effects on self-reported physical activity for housekeeping and gardening, self-reported leisure-time physical activity and self-reported total physical activity.

#### Step 3b - Mediating effects of change in modeling from family

After adjusting for change in modeling from family (Table 4), the positive intervention effects remained significant for change in self-reported leisure-time physical activity (p = .001). However, the adjusted regression coefficients (τ') were significantly lower than the mediator-unadjusted τ-coefficients (t = 2.07). This indicates that the short-term increase in modeling from family mediated the intervention effects on self-reported leisure-time physical activity (12.2%).

Change in modeling from family was not a significant mediator of the short-term intervention effects on steps/day, self-reported active transportation, self-reported total physical activity for housekeeping and gardening and self-reported total physical activity.

#### Step 3c - Mediating effects of coping with relapse

After adjusting for coping with relapse (Table 4), the intervention condition remained a significant positive predictor of change in steps/day (p < .001), change in self-reported active transportation (p = .006), change in self-reported leisure-time physical activity (p = .015) and change in self-reported total physical activity (p = .001). However, the adjusted regression coefficients (τ') were significantly lower than the unadjusted τ-coefficients (t-values from 2.05 to 2.36). Thus, coping with relapse mediated the intervention effects on steps/day (6.1%), self-reported active transportation (28.4%), self-reported leisure-time physical activity (11.9%) and self-reported total physical activity (11.3%).

Coping with relapse was not a significant mediator of the short-term intervention effects on self-reported physical activity for housekeeping and gardening.

### Changes in psychosocial factors as mediators of intermediate-term (pre-follow up) intervention effects on physical activity outcomes (Table 5)

**Table 5:** Mediating effects on the intermediate-term (pre-follow up) intervention effects on change in physical activity (PA) behaviors

	Steps/day	Self-reported active transport	Self-reported PA house + garden	Self-reported leisure-time PA	Self-reported total PA
τ (SE)	2491.98 (617.29)	51.41 (28.61)	190.78 (61.62)	55.62 (29.08)	283.46 (62.76)
p	< .001	.076	.003	.059	<.001

*Mediator: change in self-efficacy towards barriers of PA*					
τ' (SE)	2313.21 (684.59)	32.15 (32.69)	178.66 (71.05)	46.82 (32.95)	253.65 (71.46)
p	.001	.329	.014	.160	.001
τ - τ'	178.77	19.26	12.12	8.80	29.81
t	1.11	3.16*	.70	1.12	2.00*
Proportion mediated		44.3%			10.5%

*Mediator: change in social support from family*					
τ' (SE)	2331.57 (698.80)	36.24 (33.52)	146.75 (70.91)	40.21 (33.07)	226.30 (69.31)
p	.001	.283	.042	.228	.002
τ - τ'	160.41	15.27	44.03	15.41	57.16
t	1.07	2.37*	2.54*	2.16*	4.07*
Proportion mediated		29.5%	23.1%	27.7%	20.2%

*Mediator: coping with relapse*					
τ' (SE)	2419.71 (662.78)	36.83 (30.89)	205.54 (67.33)	52.28 (31.38)	277.75 (68.40)
P	<.001	.237	.003	.100	<.001
τ - τ'	72.27	14.58	-14.76	3.34	5.71
t	.55	2.06*	.96	.49	.37
Proportion mediated		28.4%			

*p < .05

τ = relationship between intervention condition and outcome measure before adjusting for mediator

τ' = relationship between intervention condition and outcome measure after adjusting for mediator

SE = standard error

*Note*: in all analyses, the total sample (n = 92, both control group and intervention group) was included

#### Step 1

After controlling for the confounding variables, the intervention was a significant positive predictor of intermediate-term change in steps/day (p < .001), self-reported physical activity for housekeeping and gardening (p = .003) and self-reported total physical activity. The intervention was a marginally positive predictor of self-reported active transportation (p = .076) and self-reported leisure-time physical activity (p = .059). The mediator-unadjusted τ-coefficients of the significant regression analyses are shown in Table 5.

#### Step 2

The intervention was a significant positive predictor of intermediate-term change in specific self-efficacy towards physical activity barriers (β = .183; SE = .089; p = .044) and of coping with relapse (β = .436; SE = .215; p = .046), and a marginally significant positive predictor of intermediate-term change in social support from family (β = .339; SE = .196; p = .088). For the different types of motivation, modeling, social norm, social support from friends and partner, general self-efficacy, benefits and barriers, no significant results were found (all p > .10). Therefore, only change in self-efficacy towards physical activity barriers, change in social support from family and coping with relapse were analyzed as potential mediators of the intermediate-term (pre-follow up) intervention effects on changes in physical activity behaviors.

#### Step 3a - Mediating effects of change in self-efficacy towards physical activity barriers

After adjusting for change in self-efficacy towards physical activity barriers (Table 5) the intervention condition remained a significant positive predictor of change in self-reported total physical activity (p = .001). However, the adjusted regression coefficient (τ') was significantly lower than the mediator-unadjusted τ-coefficient (t = 2.00). This shows that the intermediate-term increase in self-efficacy towards physical activity barriers mediated the intervention effect self-reported total physical activity (10.5%).

A second mediating effect of change in self-efficacy towards physical activity barriers was found for the intermediate-term increase in active transportation. For this variable the intervention effect became insignificant (p = .329) and the adjusted regression coefficient (τ') was significantly lower than the unadjusted τ-coefficient (t = 3.16). Thus, the intermediate-term increase in self-efficacy towards physical activity barriers mediated the intervention effect on self-reported active transportation (44.3%).

Change in self-efficacy towards physical activity barriers was not a significant mediator of the intermediate-term intervention effects on steps/day, self-reported leisure-time physical activity or self-reported physical activity for housekeeping and gardening.

#### Step 3b - Mediating effects of change in social support from family (Table 5)

After adjusting for change in social support from family, the positive intervention effects remained significant for change in self-reported physical activity for housekeeping and gardening (p = .042) and change in self-reported total physical activity (p = .002), but the adjusted regression coefficients (τ') were significantly lower than the unadjusted τ-coefficients (t = 2.16 and t = 4.07). This indicates that the intermediate-term increase in social support from family mediated the intervention effects on self-reported physical activity for housekeeping and gardening (23.1%) and self-reported total physical activity (20.2%).

Two other mediating effects of change in social support from family were found for the intermediate-term increase in active transportation and in self-reported leisure-time physical activity. For these variables the intervention effect became insignificant (p = .283 and p = .228) and the adjusted regression coefficients (τ') were significantly lower than the unadjusted τ-coefficients (t = 2.37 and t = 2.16). Thus, the intermediate-term increase in social support from family mediated the intervention effect on self-reported active transportation (29.5%) and on self-reported leisure-time physical activity (27.7%).

Change in social support from family was not a significant mediator of the intermediate-term intervention effects on steps/day.

#### Step 3c -Mediating effects of coping with relapse

After adjusting for coping with relapse (Table 5), the intervention effect on self-reported active transportation became insignificant (p = .237) and the adjusted regression coefficient (τ') was significantly lower than the unadjusted τ-coefficient (t = 2.06). This indicates that coping with relapse mediated the intervention effect on self-reported active transportation (28.4%).

Coping with relapse was not a significant mediator of the intermediate-term intervention effects on steps/day, self-reported physical activity for housekeeping and gardening, self-reported leisure-time physical activity or self-reported total physical activity.

## Discussion

The aim of this study was to determine whether the intervention effects on physical activity found in our pedometer-based telephone supported, behavioral modification intervention were mediated by the theoretical constructs targeted by the intervention (e.g. self-efficacy, social support, motivation, coping with relapse). Post-intervention (short-term) and one-year (intermediate-term) psychosocial mediators of the changes in either objective or self-reported physical activity were investigated in a randomized controlled trial. In line with the hypothesis, the results revealed that some changes in psychosocial constructs mediated the intervention effects on physical activity. However, mediators were different at the short or intermediate term and highly dependent on the measure of physical activity.

Coping with relapse, defined as the ability to avoid and cope with relapse-inducing situations, was the most consistent mediator over the short-term. During the intervention, participants learned how to cope with future high-risk situations; coping with relapse and getting active again after a period of relapse was a frequent theme in the telephone calls with patients. Six months after the end of the intervention, the ability of patients to cope with relapse still mediated 28.4% of the intervention effect on self-reported active transportation. There are no physical activity studies with which we can compare this effect. Coping with relapse is seldom measured, and was never included in mediation analyses to explain intervention effects on physical activity. Nonetheless, in a study examining possible mediators of the effectiveness of a smoking cessation program, coping with relapse was identified as a significant mediator of the short-term effect of the cessation program [40]. Moreover, in clinical practice and also in the Transtheoretical Model [7] it is considered to be a major factor in sustained behavior change. Although it might be too early to draw firm conclusions on the role of coping with relapse as an important mediator in explaining intervention effects in diabetes patients, this construct should be included in further studies and probably also be part of intervention strategies to increase physical activity in diabetes patients.

General self-efficacy was most often found to be a major mediating factor in previous studies on mediators of physical activity in the general population. In the present study, not general self-efficacy, but changes in self-efficacy towards overcoming specific physical activity barriers was an intermediate-term mediator, mediating 44.3% and 10.5% of the intervention effect on active transportation and total physical activity, respectively. Other intervention studies have reported clear effects on specific self-efficacy (barrier and task self-efficacy, self-efficacy under specific circumstances and in specific difficult situations) both in a general and type 2 diabetes populations, however only over the short-term [16,19,24,41]. In our study, self-efficacy towards physical activity barriers was not a mediator during the intervention period (short-term), but only after the intervention ended (intermediate-term). This finding is in line with the theory of Marlatt and Gordon [39] suggesting that individuals who initiate behavior change, experience increased self-efficacy that grows as they continue to maintain the change. This reciprocal relationship between behavior and self-efficacy might explain the fact that specific self-efficacy became only a mediator of behavior change after a certain period of intervention. This finding also implies that interventions that can increase patients' self-efficacy towards physical activity barriers seem to be particularly important for maintaining physical activity changes over the intermediate-term [39]. The intervention did not succeed to have a significant impact on general self-efficacy towards physical activity. One reason could be that general self-efficacy was queried to vaguely. This pleads for including specific self-efficacy in future studies.

In addition to the mediation effects found for coping with relapse and self-efficacy towards barriers, a third group of mediators was found, all related to social factors. Increases in social norm and modeling from family mediated some of the short-term intervention effects. This underlines the need for an environment with physically active family members (modeling) who have clear physical activity expectations towards the participant (social norm). In the initial face-to-face session, modeling was discussed and most of the participants' spouses were present, which may have increased their physical activity expectations towards the participant. Our results supported the early emphasis on modeling to yield especially changes in leisure-time activities. These activities were often performed together with a partner or a friend, which means that modeling can be interpreted here as being active together with a 'sportpartner'. Increases in social norm from family were mainly related to increases in steps and active transport, which means that the intervention succeeded in changing the perceptions of partners of the patients and in attempts to encourage them to take steps or to walk or cycle for transport. Social support from family did not mediate short-term physical activity changes but was the most consistent mediator of intermediate-term changes of physical activity. An explanation for this effect could be that the participants received enough support from the study psychologist during the intervention and did not rely on their family for further support. Unfortunately, the perceived support from the psychologist was not queried by any of the questionnaires. After the intervention, however, participants did rely again on their family for support. Barrera and colleagues [23] also found a mediating effect of social factors in type 2 diabetes patients but only over the short-term, during the 6 months intensive intervention period. However, in that study a general social resources measure was used summing the effects of family, friends and neighbors and not distinguishing between specific constructs such as social norms, modeling or support, which makes comparison with our effects rather difficult. No other studies examining the mediating role of social factors on physical activity changes in type 2 diabetes patients were found. However, in general adult populations, social factors like social support do not appear to mediate intervention effects on physical activity [42-44]. Possibly, social factors are of higher importance in specific populations like diabetes patients than they are in the general adult population.

There was no increase in autonomous motivation (i.e. introjected regulation, identified regulation, intrinsic regulation) towards physical activity in this study group, although our intervention also incorporated self-determination theory constructs. An explanation could be that it is very difficult to intrinsically motivate this elderly chronically ill population. Because of their age and illness, they seemed to be more externally motivated to be physical active (because it is good for their health, because they need to take less medications, etc.). An alternative explanation could also be that the content of the intervention or the delivery mode did not support autonomous motivation adequately. Secondly, our intervention did not succeed in increasing the benefits and decreasing the barriers of physical activity, despite the fact that part of the face-to-face session and the telephone sessions with the patients were dedicated to these constructs, and these constructs are considered to be important mediators of behavior change in several popular models or theories [6-8]. If future studies confirm that it is hard to change motivation, barriers, and benefits of physical activity, and these constructs do not act as mediators to change physical activity, a possibility could be to delete these elements from the intervention, in order to make the intervention more restricted and to have a stronger focus on a limited number of constructs. This was suggested before in a study examining mediators of a physical activity intervention in adolescents [45]. It is however important to notice that self-efficacy towards physical activity barriers and coping with relapse were found to be important mediators, so listing personal physical activity barriers could possibly be seen as a first step needed to increase self-efficacy and decrease relapse. Therefore, before concluding that certain constructs (e.g. perceived barriers and benefits) could be deleted from interventions, one needs to know whether there is a possibility that they have an indirect effect on physical activity. Further research on this subject is needed.

In line with the recommendations of King and colleagues [9], we tried to gain a wider understanding of mediators across different physical activity domains in the present study. As mentioned before, most constructs only mediated effects on specific measures of physical activity. Although the intervention had a strong impact on physical activity for housekeeping and gardening, few mediating effects were found (only social support on the intermediate-term). A possible explanation can be that gardening and housekeeping are tasks that have to be done routinely, and are thus the result of unconscious individual decision-making [46]. It is possible that just by wearing a pedometer, through monitoring their behavior, people increase their gardening and housekeeping [47].

The results of this study should be viewed in light of its limitations. The physical activity intervention was delivered from a tertiary care-based setting, which may not generalize to other community samples or settings. Results might differ if participants were recruited from community or primary care settings. Secondly, although previous studies have shown that self-reported psychosocial measures have good reliability and acceptable validity, they could suffer from social desirability [35]. Thirdly, only 92 diabetes patients participated in this study. Because of this limited sample size, also marginally significant results were included in this paper. If future studies would include a larger study sample, stronger findings could possibly be identified.

Despite these limitations, the present study is noteworthy given the very limited number of studies examining mediating effects of a physical activity intervention in type 2 diabetes patients. A second strength is that the present study also investigated intermediate-term mediators, after a one-year period. Because of the overall effectiveness of this randomized controlled trial on physical activity measurements, this is one of the first true mediation analyses in a type 2 diabetes sample.

## Conclusion

Our findings indicate that coping with relapse can be an important mediator of changes in different types of physical activity during the 6 month intervention period (short-term), while social support is a mediator of change in the longer term. Self-efficacy towards overcoming specific physical activity barriers was found to be an additional intermediate-term mediator. Positive social norms and modeling from family were significant initial mediators of physical activity change.

Future interventions should give particular attention to teach participants how to cope with high-risk situations, to train their skills and self-efficacy to overcome physical activity barriers, and to mobilize family members to support them to be active or to engage in physical activity together with them. As this is one of the few studies focusing on mediators of change in a physical activity intervention for adults with type 2 diabetes, additional research is necessary to confirm and extend these findings.

## Competing interests

The authors declare that they have no competing interests.

## Authors' contributions

All authors contributed to the design of different parts of the study. KDG designed the psychosocial scale, was responsible for data acquisition, carried out the intervention and drafted the manuscript. DVD performed the statistical analyses and drafted the methods and results sections of the manuscript. BD and IDB participated in the interpretation of the data and helped to draft the manuscript, revised the manuscript for important intellectual content and supervised KDG as part of their PhD training. DVD, JR, CTL and JMK revised the draft for important intellectual content. All authors read and approved the final manuscript.
